# Contextualizing assessment feedback in translation education: A corpus-assisted ecological approach

**DOI:** 10.3389/fpsyg.2022.1057018

**Published:** 2022-12-19

**Authors:** Deliang Man, Chenghao Zhu, Meng Huat Chau, Elanttamil Maruthai

**Affiliations:** ^1^Center for Translation Studies, Guangdong University of Foreign Studies, Guangzhou, China; ^2^Faculty of Languages and Linguistics, Universiti Malaya, Kuala Lumpur, Malaysia

**Keywords:** ecological perspective, translation assessment, translation education, sociocultural theory, dynamic assessment, longitudinal translation corpus

## Abstract

Despite its powerful influence on student learning, assessment feedback has received relatively less attention in translation education. The mainstream assessment practices in translation education have relied mainly on a static approach to translation competence. The consequences of a static approach include a partial representation of translation competence development and a deficit view of students and their learning. Alternatively, this paper argues for an ecological approach to contextualize assessment feedback in translation education. The ecological approach emphasizes the spatial and temporal context for translation assessment. While detailed contextual information is essential to the ecological assessment approach, assessing translation performance across tasks and time is a considerable challenge. In response to such a challenge, this conceptual paper proposes a corpus-assisted approach to translation assessment. It discusses how a longitudinal student translation corpus can be developed to assist ecological assessment feedback on translation performance. A project in progress based on a translation education program is reported as a case in point for illustrative purposes. The paper has suggested ways forward for future assessment feedback practice and research in translation education.

## Introduction

Assessment feedback has been recognized as a powerful influence on student learning (Hattie and Timperley, 2007). It has been shown that proper assessment can promote student learning, whereas improper assessment can inhibit student learning (Wiliam, 2011). Despite an increasing body of research on assessment feedback in areas such as second language (L2) writing (Ruegg, 2015; Falchikov and Goldfinch, 2016; Double et al., 2019; Yang et al., 2021), relatively few studies have investigated assessment feedback in translation education. This lack of research is surprising, considering the centrality of assessment feedback in translation education (e.g., Albir and Pavani, 2018; Shorofi et al., 2020). Assessment of translation is different from L2 writing assessment. It involves judgment of the level of correspondence between the source text and the translation (Han and Lu, 2021). In the few studies on translation assessment (e.g., Yu et al., 2019; Ge, 2022; Li and Ke, 2022), assessment feedback centered on translation tasks and was implemented following the traditional static approach that focuses on current or actual levels of competence for summative assessment purposes (Haywood and Lidz, 2007). In these studies, student performance was often treated with the same assessment standard across tasks and time in the name of test validity and reliability. This seeming objectivity has, however, been questioned from a sociocultural perspective on learning (van Lier, 2004; Lantolf and Poehner, 2013; Poehner and Wang, 2020). It has been argued that students do not develop their abilities at the same pace, and their performance is subject to the environment where they perform the specific task (van Lier, 2004; Larsen-Freeman, 2019). Even if students have the same difficulty, they might require different forms of support at different points in time (Aljaafreh and Lantolf, 1994). However, the existing approaches to translation assessment, with their focus on validity and reliability, often ignore individual differences and their varied performance across contexts.

This paper proposes an ecological approach to assessment feedback as a contextualized practice in translation education to support student development. The paper is inspired by ecological approach to language learning of van Lier (2004). The ecological approach highlights the relationships between learners and their surrounding environment and considers learners as agentic participants of activities in their spatial and temporal context. It is change-oriented and critical of the implications for research and practice. While the ecological approach has the potential for translation assessment, it remains unclear how assessment feedback can be practiced following this ecological approach. This conceptual paper seeks to address two questions crucial to ecological assessment feedback:

1. Is it possible to access the information required to evaluate students’ translations in their spatial and temporal context? And how?
2. How can an ecological approach be integrated into assessment feedback in translation education?

In answering these questions, this paper reports on a project in progress that aims to develop a corpus-assisted approach that gathers rich contextual information for practicing ecological assessment. The proposed design of a student translation corpus and the accompanying analytical tools under construction are presented to describe how this corpus-assisted approach can be implemented in a translation education program. The paper has suggested ways forward for future assessment feedback practice and research in translation education.

## An ecological perspective on language learning

The ecological perspective on language learning is not “a grand theory” but a coherent and consistent worldview based on ideas, practices, and evidences from different theoretical orientations (van Lier, 2004). One prominent theoretical orientation is the sociocultural theory (SCT), an overall approach to human sciences whose goal is to explain the relations between mental functioning and the cultural, institutional, and historical situations in which this functioning occurs (van Lier, 2004). SCT conceptualizes language learning as a mediated process: learning is mediated through various forms of physical and symbolic tools, artifacts, and social interactions (Lantolf, 2000). From an SCT perspective, there is a fundamental difference between learning and development. Learning occurs when learners can complete a task with external mediation, while development takes place when learners can self-regulate without external mediation (Lantolf, 2000). Learning reflects the abilities that have begun to emerge but have not yet fully developed, whereas development indicates those that have fully developed at the time of the assessment. Thus, the assessment of learning involves a consideration of an individual’s responsiveness to mediation, such as reminders, hints, models, and feedback provided during the assessment as difficulties arise (Poehner and Wang, 2020). Assessment of development, on the other hand, is performed when an individual independently completes a task. A second theoretical approach is the complexity theory perspective on language learning (Larsen-Freeman, 1997, 2011). Complexity theory views language as a dynamic set of patterns emerging from use. Language learning emerges when relatively simple elements combine to form a higher-order system that is more than the sum of its parts.

Drawing on these theoretical orientations, van Lier (2004) proposed a set of criteria for the application of an ecological perspective to research and practice (p. 193):

1. It is contextualized, focusing on relationships in the setting.
2. It includes both spatial and temporal dimensions.
3. It is interventionist, i.e., change-oriented and critical.
4. It is ecologically valid, particularly in correspondence between researchers’ and participants’ situation definitions.

The ecological perspective contextualizes learning and development. It conceptualizes language as relations between people and the world and language learning as ways of relating more effectively to people and the world. These relations provide an affordance that signals an opportunity for or inhibition of action. The unit of learning is the learner in action in a learnable environment. Language learning is an area of activities, and learners develop by participating in these activities. The context for language learners is not just something that surrounds language, but that defines language‚ and, at the same time, is defined by it. Learners are considered autonomous and agentic individuals who can define the meaning of their acts within their social context.

The ecological perspective considers not only factors of space (e.g., the physical, social, and symbolic affordances of the learning environment) but also the temporal dimension for learning and development. It postulates that language learning is not gradual‚ linear acquisition‚ but emergence, which happens when relatively simple elements combine to form a higher-order system, as noted above. The new system is on a different scale‚ and has different meanings and patterns of functioning than the simpler ingredients from which it emerged. The complexity of development over time inevitably results in variability in the paces and trajectories of learning. The ecological approach addresses this variability issue not by treating learners all the same but considering the many differences among learners relevant to their learning opportunities. Given the vast diversity among learners, the ecological perspective embodies the value of diversity, recognizing the heterogeneity of the population of learners, and giving legitimacy to having different learners in the same educational setting and different kinds of people in a society. It is worth noting that the temporal dimension extends the notion of variability from the individual differences in students’ formed abilities to the paths and paces of their development.

Language teaching and research from an ecological perspective are change-oriented and critical. Such a perspective aims to introduce a change, fix a problem, or transform a piece of reality. Education’s primary objective is to benefit students’ lifelong careers and well-being. However, what we consider worthwhile outcomes are not free from values. The consideration of values is referred to as a critical perspective in the ecological approach, defined as “any approach (scientific or otherwise) to a state of affairs that applies an explicit and overt rational, moral and ethical stance to the treatment, interpretation, and documentation of that state of affairs” (van Lier, 2004, p. 168). The ecological approach considers all actions and practices as value-laden‚ value-driven, and value-producing. From an ecological perspective, language education is always a science of values. In other words, educational assessment is not necessarily about objective facts. A critical perspective on language education calls for a constant examination of the extent to which educational practices, including assessment, make for the specific goals and ideals that have been established and pursued. One educational ideal is to benefit the students and enhance their well-being (e.g., Chau et al., 2022). However, the so-called high education standards do not necessarily lead to good results or pleasant educational experiences because the quality of educational experience is differentiated by educational standards.

The final criterion is ecological validity. According to Bronfenbrenner (1979), ecological validity is “the extent to which the environment experienced by the subjects in a scientific investigation has the properties it is supposed or assumed to have by the investigator” (p. 29). In other words, the analytic notions and constructs researchers and teachers use should correspond to those used by participants and students in the same setting. In the case of language teaching and learning, teachers and students should have the same understanding of language and language learning and the same metalanguage to talk about these two things.

Drawing on the ecological approach to language learning, language researchers have developed an ecological perspective on assessment feedback (e.g., Han, 2019; Chong, 2021; Man et al., 2022). The ecological perspective emphasizes the role of contextual factors and individual differences in shaping feedback practices. The context includes a range of temporal, spatial, material, and social factors (Gravett, 2022), and the individual differences concern learners’ proficiencies, goals, beliefs, and experiences (Chong, 2021). While this ecological perspective has found its way into assessment feedback research, relatively few studies have considered how to apply this ecological perspective in practice. To date, the assessment approach reported in research on Dynamic Assessment (henceforth DA) can be called ecological, though without explicit use of the term. The DA approach emphasizes the context. It considers the mediation learners receive in their developmental process and conceptualizes mediation as a means to close the gap between the actual and potential levels (Vygotsky, 1978). The distinctive feature of DA from static assessment is that it is committed to extending observations from an individual’s independent task performance to collaborative performance with others (Lantolf and Poehner, 2013). DA in the language classroom has two functions: (1) to assess mediated learning performance and (2) to promote student learning through mediation. In other words, mediation is not only meant to scaffold individuals to complete tasks but also to help determine the minimum level of support they need for successful completion (Lantolf and Poehner, 2013).

While the ecological perspective has received considerable attention in language education research (Kramsch, 2002; Han, 2019; Poehner and Wang, 2020; Chong et al., 2022; Man et al., 2022), only a handful of studies have attempted to apply an ecological approach to translation education. For example, in a conceptual paper, Mo and Man (2017) discussed how an ecosystem view could inform the design of an environment for the cultivation of technological competence. A more recent study by Shorofi et al. (2020) examined the effect of Group Dynamic Assessment on student translators’ bilingual sub-competence development. While these studies have revealed the value of an ecological perspective for translation education, little is known about how the ecological perspective can be applied to the assessment of translation performance. As Han and Lu (2021) have pointed out, the assessment of translation competence requires evidence-based judgment of translation performance. The accumulating research on translation education using an ecological perspective needs to move beyond the macro level to the micro level of translation performance assessment.

## Toward an ecological approach to translation assessment

Developing students’ translation competence is central to translation programs. The literature has documented multiple methods of assessing translation competence (Schäffner and Adab, 2000; PACTE, 2014; Albir et al., 2020). For example, one approach that has been repeatedly used, though mostly in experimental research, is to triangulate multiple sources of data (e.g., students’ screen recordings, reflective journals, and questionnaire responses; e.g., PACTE, 2014; Albir et al., 2020). Recent endeavors have also aimed to develop level descriptors that describe and measure translation competence levels (e.g., Bai et al., 2018; PACTE, 2018). While these methods consider both the translation products and the translation process, the considerable time involved in triangulation often determines that the assessment can only be conducted a limited number of times for a relatively short period. Given the non-linear developmental path of student learning (Larsen-Freeman, 2011, 2019) and the diverse contextual factors that could influence the development (van Lier, 2004; Chong et al., 2022), evaluation of students’ translation competence must cover a relatively long period, with full attention to the spatial and temporal context wherein translation is produced.

This paper proposes an ecological approach to assessment feedback in translation education. From an ecological perspective (van Lier, 2004), translation competence develops in a non-linear manner and emerges from learners’ interaction with and within their context (Kiraly, 2015). Student learning is measured by their efforts to complete translation tasks with external mediation, such as technical assistance, peer feedback, and teacher support (Lantolf, 2000). In contrast, development is evidenced by the closure of the gap between the actual level reflected in independent task performance and the learning potential reflected in collaborative performance supported by external mediation (Lantolf, 2000). Therefore, this ecological approach to assessment feedback involves considering translation performance across tasks and time. By doing so, assessment feedback recognizes students’ learning efforts and internalized development.

The four criteria of van Lier (2004) recommended are considered in translation assessment: contextualized relationships, spatial and temporal dimensions of context, change-oriented, and ecological validity. In the first place, ecological assessment feedback considers the contextual factors and supports using environmental affordances to foster student learning and development. Second, the evaluation of learning progress considers the external mediation (e.g., teacher feedback, peer support, and technical assistance) that students receive before, during, and after their translation task. The development is also considered on a temporal dimension to account for variability across time. Third, the purpose of assessment feedback is to bring about change in students’ translation learning. The influence of the implemented assessment is critically and continuously evaluated to determine if the original intentions are fulfilled. Finally, the assessment criteria are aligned with students’ conceptions of learning and development, or at least the assessment criteria are explained to students to ensure correspondence between assessors’ and students’ definitions.

Despite their potential, implementing the four criteria of the ecological perspective is not without challenges. The immediate challenge in assessing translation performance in its spatial and temporal context is to access and compare a wide range of information on learner data across tasks and time. The diversity and variability further complicate this challenge in learning and development, reflected by a wide range of behavioral performances and measured by a diversity of indicators such as holistic human ratings, linguistic analyses, and translation errors. Given the large number of students and the many thousands of words of translation they must complete as part of their program objective, the workload that arises from sorting out all this information for assessment is formidable. Technological affordance is desired to assist this assessment process.

Corpus linguistics is a powerful tool to assist the storage, retrieval, and analysis of translation texts produced across tasks and time. While the idea of building up a dataset of longitudinal student translator data is not new, longitudinal corpora of student translation are still very rare (Göpferich, 2013). The existing student translator corpora are at best cross-sectional (Espunya, 2014; Alfuraih, 2020; Granger and Lefer, 2020). In the following section, we will discuss a longitudinal database of student translation in construction for ecological assessment purposes. We report on this project in progress for illustrative purposes.

## Constructing a longitudinal corpus of student translation

What we call a database is an accumulating archive of data that includes a variety of information in addition to student translation. These include the source texts to be translated, the feedback that students’ translations will receive, and the descriptions of the translation processes.

### Principles and procedures

Student translation corpora can be considered two-in-one resources, as they contain systematic collections of authentic and contextualized written translations produced by trainee translators (Granger and Lefer, 2020). According to Sinclair (2005), the most relevant principles for developing a corpus are:

1. Representativeness: select participants and contents to be as representative as possible.
2. Content selection: select corpus contents based on external criteria (communicative function of the texts) and not internal criteria (the language of the texts).
3. Topic: select the subject matter of the corpus based on external criteria.
4. Contrast: compare only those sub-corpora that have been equally designed.
5. Documentation: fully document the contents of the corpus (i.e., metadata).

The student translation corpus to be built combines a parallel corpus (Baker, 1995) and a learner corpus (Granger and Lefer, 2020). Thus, the corpus construction needs to consider the principles of these two types of corpora. In addition, as the corpus under construction, in the present study is to assist ecological assessment feedback on students’ translation competence development, the corpus will contain and provide the following information:

1. Metadata about the translation texts.
2. Information about the source texts to be translated.
3. Information about external mediation in the learning progress (e.g., feedback).
4. Spatial and temporal contextual information on translation performance.
5. Illustrated assessment criteria aligned with students’ conceptions of learning and development.

**Figure 1:**
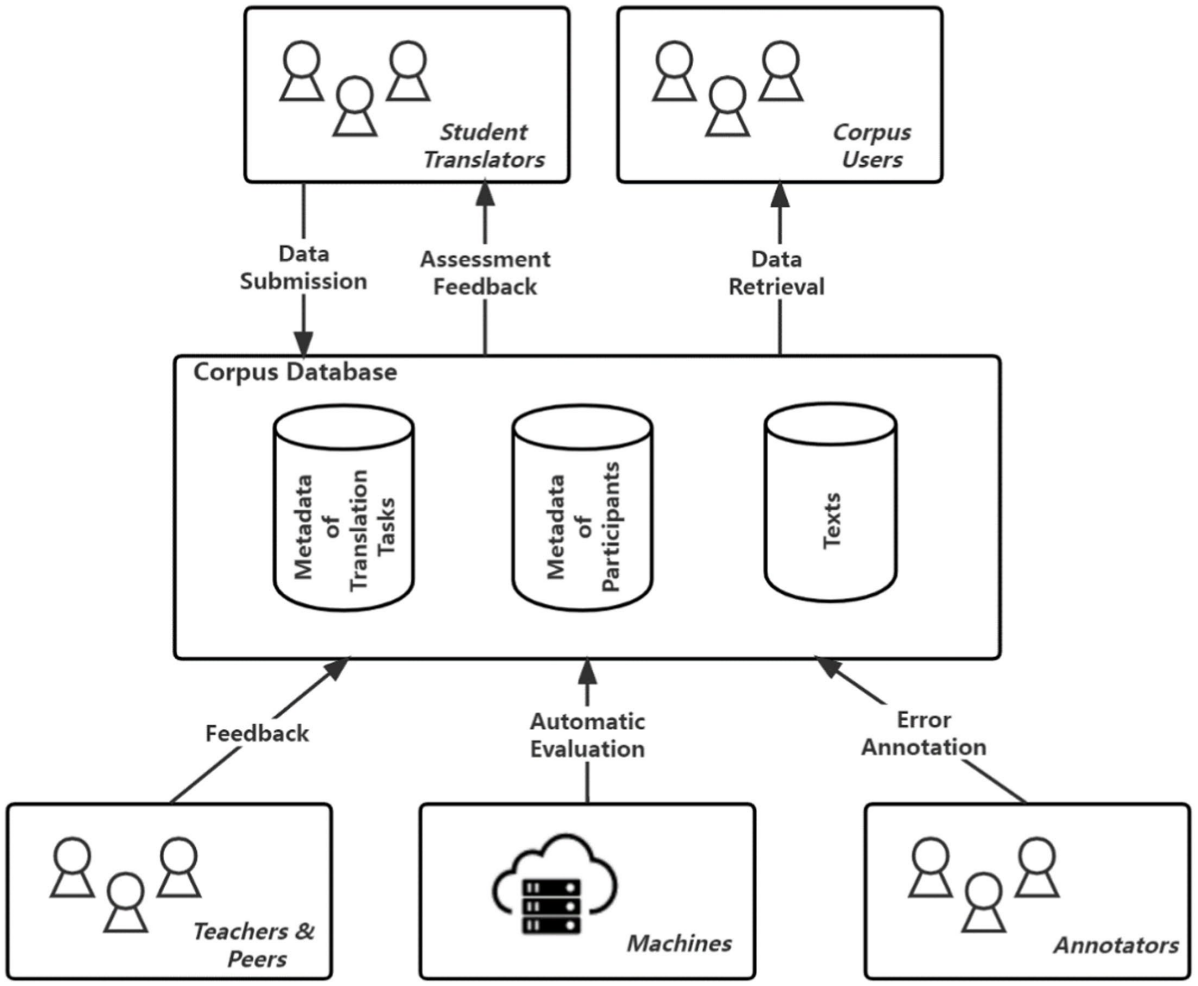
Procedures of corpus construction.

Following these principles for corpus construction, we have designed a set of procedures for data collection. It should be noted that the data will be collected from students of a translation education program through an online platform. Figure 1 presents the procedures of data collection and retrieval. More details will be presented in the section on corpus design.

### Data collection

This ongoing project is based on a 2-year master’s program in translation at a Chinese university. This program enrolls around 120 students each year, and students must complete translation practice of a minimum of 150,000 words between Chinese and English before they defend their dissertation. During their translation practice, students choose the translation directions and materials at their discretion and then complete the translation exercise with the assistance of translation tools and resources. Once the online platform is ready, students will be required to submit their translations and the original texts to the platform at different points in time throughout their 2 years of study. The submitted translation products will be archived in a principled manner to form a longitudinal student translation corpus. The students will also receive feedback from various sources (i.e., teacher feedback, peer feedback, and computer-generated feedback) on their translation performance. Such feedback information reflects students’ developing translation competence and indicates the learning potential of students to complete translation tasks independently.

### Corpus design

All the texts will be submitted to the online platform with rich metadata. These include the topic, genre, source of source texts, conditions under which the translation is produced, and demographic information on the translators, annotators, and feedback givers. More details about the metadata are presented as follows.

#### The metadata of source texts

The metadata of the source texts provides a wide range of information. These include Source Text ID, Language (i.e., Chinese or English), Title, Genre, Topic, and Language Type (see Table 1). General language comprises texts written in a non-technical language, such as journalistic and fiction texts. In contrast, specialized language comprises texts written in a technical language, such as academic prose and instructional texts (instructions and manuals; Granger and Lefer, 2020). A set of topics are provided to allow students to label their source texts (e.g., Education, Entertainment, and Finance). Students can also add topics if the default topic tags do not describe their source texts. Students provide information such as title, topic, and language type, and other information, such as Length (i.e., the number of words or characters), Timestamp, and Source Text ID are automatically recorded in the system.

**Table 1:** Metadata for source texts.

Metadata	Description
Source Text ID	
Timestamp	00:00:00, dd-mm-yyyy
Language	English, Chinese
Text title	______________
Language Type	General/Specialized
Genre	General (e.g., journalistic texts and fiction) Specialized (technical report and academic prose)
Topic	Education, Entertainment, Finance, etc.
Length	Word/character number

#### The metadata of target texts

The metadata of target texts records details about the translation tasks and the translation products (see Table 2). Some of the metadata information can be presented by students when they submit their translation, for example, Task Type (e.g., an in-class activity or a home assignment), Translation Direction (i.e., Chinese to English or English to Chinese), Task Duration (e.g., timed, untimed, or duration in minutes), and Tools and Resources used in the translation task. The computer can automatically generate other information, such as word counts. Because teachers and fellow students can grade or comment on the translation through the online platform, ratings and feedback comments are archived along with the translation and its original texts.

**Table 2:** Metadata for the translation process.

Metadata	Description
Target Text ID	
Timestamp	00:00:00, dd-mm-yyyy
Type of Task	In-class activity, examination, home assignment, voluntary exercise, and mixed
Duration of Task	Timed, untimed, and duration in minutes
Tools and Resources	Monolingual dictionaries, bilingual dictionaries, glossaries, and terminological databases, translation forums, bilingual concordancers, SL and TL general or specialized corpora, CAT tools with TDB, CAT tools with TM, CAT tools with both TDB and TM, machine translation, internet/web
Rate	Yes/No
Annotation	Yes/No
Length	Word/character number

#### The metadata of students, instructors, and annotators

Informed consent will be obtained from students and teachers before their particulars are recorded on the platform. Identifying information will be kept to a minimum to protect the users’ privacy. The required information includes gender, age, school year, educational background, and working experience (see Table 3).

**Table 3:** Metadata for students.

**Metadata**	**Description**
Student ID	
Gender	Male, female
Age	
Grade	e.g., first year, second year
Prior Education Background	Majors: e.g., English, medicine, and engineering
Working Experience	Yes/No
Number of Years Learning English	
Whether or not they stayed in an English-speaking country	Yes/No
Self-rated English proficiency	Intermediate, advanced, and native
Test-based English proficiency	CET/TEM/IELTS/TOFEL scores

Information about potential feedback sources is also included. In line with previous studies (e.g., Alfuraih, 2020), information about teachers and program administrators who provide feedback is collected (see Tables 4, 5). This metadata information provides a context for interpreting the feedback and assessing the level of external mediation.

**Table 4:** Metadata for teachers and program administrators.

**Metadata**	**Description**
Instructor ID	
Gender	Male, female
Age (years)	
Major	
Degree	Bachelor, Master, PhD
Teaching experience	

**Table 5:** Metadata for Annotator.

Metadata	Description
ID	
Gender	Male, female
Age (years)	
Grade	e.g., first year, second year
Major	

#### Corpus annotation

The corpus will be coded and annotated to allow multiple ways of text analysis (Hunston, 2002). Firstly, the English texts in our corpus will be pre-processed with word tokenization, lemmatization, and POS tagging, whereas the Chinese texts will be processed with word segmentation and POS tagging. The Stanza, a computational linguistic toolkit for many human languages developed by Stanford NLP Group (Qi et al., 2020) will be employed for these purposes. Second, part of the corpus will be coded for translation errors. A module will be designed, and taxonomy will be provided for manual annotation of translation errors. The module allows researchers or teachers to customize the taxonomy of translation errors for annotation purposes (e.g., Espunya, 2014; Zhao et al., 2018; Granger and Lefer, 2020; Qian et al., 2022). The advantage of the module is that it is open, and the coding system will be developed and revised iteratively as the annotation process proceeds.

#### Metrics from machine translation systems

Manual evaluation of a large amount of translation data produced by students takes considerable time, even with the assistance of corpus tools. However, students often expect immediate feedback after they complete a translation task. A possible means to fulfill such expectations is to produce computer-generated feedback using machine translation systems. Recent studies have shown the potential and feasibility of machine translation metrics to evaluate student translation (Chung, 2020; Han and Lu, 2021). When electronic texts are archived and accessible through a computer, machine translation metrics can be automatically generated to assist in analyzing the translation products. The generation of feedback from automatic translation evaluation requires only the input of one or more reference translations as the baseline for evaluating the quality of candidate translation texts.

The machine translation metrics used to evaluate student translation in the present study include such metrics as Bilingual Evaluation Understudy (BLEU; Papineni et al., 2002), Metric for Evaluation of Translation with Explicit ORdering (METEOR; Lavie and Agarwal, 2007), Translation Edit Rate (TER; Snover et al., 2006), and BERTCORE (Zhang et al., 2019). BLEU and METEOR are two algorithms based on a generalized concept of *n*-gram matching. They calculate scores to measure the similarity between a candidate translation text and the reference text(s) purposely chosen for comparison. The scores fall between zero and one, with values closer to zero representing less similar texts. TER is also a metric that can be used to assess the similarity between a candidate text and a reference text. However, unlike BLEU and METEOR scores, TER scores are measured by counting the number of edits (e.g., insertions, deletions, shifts, and substitutions) required to transform a candidate text into a reference text. A smaller TER score shows a higher similarity between the two texts. In addition to the three primary metrics, a neural network-based metric, BERTCORE, will be used in the present corpus. BERTCORE has recently been shown to correlate better with human evaluations than the three primary metrics (Zhang et al., 2019). Its calculation relies on pre-trained BERT contextual embeddings (Devlin et al., 2018) to first encode candidate and reference sentences and then compute their cosine similarity. Together all four metrics above serve as immediate feedback for students. The four metrics measure the correspondence between student translations and reference translation texts, complementing human ratings and translation error annotations. The students, teachers, and program administrators can choose the reference texts according to their purposes.

The online platform under development will feature an interface that connects the corpus and an automated evaluation system. When a piece of translation needs automated evaluation, the interface will employ the designated algorithms (e.g., BLEU and METEOR), compare the translation under evaluation and the reference translation, and produce a holistic score. The reference translation has two sources. One is from machine translation, when students select the texts to be translated by their own choice. Because the platform does not include a ready-made reference translation, API (Application Programming Interface) for machine translation (e.g., Google Translate and DeepL) will be exploited to produce a translation as the reference. In a second scenario, students translate a task designated by the teacher. A model translation will be provided as the reference translation.

### Data retrieval

This corpus features a simple syntactic expression formula that allows the combined use of Chinese character strings, English word strings, POS symbols, wildcard characters, and set symbols (see Table 6). The rules of corpus search are compatible with mainstream corpora such as the *Corpus of Contemporary American English* and *The British National Corpus* but are kept to a minimum level of sophistication. An interactive retrieval interface is also included to allow users to filter search results by metadata such as genre and topic. Users can set the number of concordance lines per page in the display settings. All retrieved concordances can be saved and exported to a designated format (e.g., excel document) for later use.

**Table 6:** Query syntax.

Type	Explanation	Examples
String	Chinese character string or English word string. Chinese character strings require no segmentation.	“make a promise” “许下承诺”
POS symbol	POS tag for each word	“V. a promise” “V. 承诺”
Wildcard character	Placeholder represented by a single character	“make a _” “许下_”
Set symbol	Within the symbol “[],” multiple strings of characters or words, separated by a “/,” indicating that they can correspond to any of the items in brackets.	“[make/have] a promise” “[许下/作出]承诺”

## A corpus-assisted approach to ecological assessment in translation education

The following sub-sections discuss how an ecological perspective can inform assessment feedback in translation education and how the corpus can be used to assist ecological assessment feedback. Assessment feedback has two purposes: (1) to assess the learning and development of students and (2) to support student learning and development. The former is often called *assessment of learning* and the latter *assessment for learning* (Wiliam, 2011). How the corpus-assisted approach to ecological assessment can fulfill these two purposes is explained below.

### Corpus-assisted ecological assessment of learning

An ecological approach emphasizes the temporal and spatial context for assessment feedback. From this perspective, the assessment results reflect students’ learning potential and already achieved development, look beyond learner-independent performance, and consider task performance with external mediation (Poehner, 2011). In line with the ecological approach, assessment feedback in translation education entails close attention to three factors: context, variability, and diversity.

The longitudinal student translation corpus provides a context for assessing student translation performance. This context includes temporal and spatial dimensions. The temporal dimension traces the changes in the various indicators of student performance, including human ratings, feedback comments, and computer-generated evaluation metrics. In this sense, the performance at a specific time has its temporal context. The spatial dimension reveals the conditions under which a piece of translation is produced. The metadata would reveal the genre and difficulty of the source text, the use of translation tools and resources, and the feedback from peers, teachers, or technology.

When student learning is examined in its temporal and spatial context, considerable variability in student performance will emerge. Students vary from each other in their translation performance at different points in time and in their own developmental path. Program administrators can assess the effectiveness of the program by measuring changes in student performance from their enrollment to their graduation.

### Corpus-assisted ecological assessment for learning

In line with the ecological approach, assessment for learning is concerned with using assessment to support student learning. In this paper, learning is conceptualized as areas of activity, learners as agents of learning, and assessment feedback as one environmental affordance to promote student learning (van Lier, 2004). Assessment feedback facilitates student learning in several ways. The first way assessment feedback promotes learning is to increase learner motivation by showing them the progress they make. The graphs of the progress of individual learners can work as a guide and stimulus to action (Fuchs and Fuchs, 1986). With the assistance of computer software, the longitudinal student translation corpus can provide relevant data on students’ progress. Such positive feedback can encourage translation students and counteract the setbacks and stresses they could experience in their challenging translation practice.

Secondly, assessment feedback helps close the gap between the current level of performance and learning potential through external mediation. An ecological perspective emphasizes the complex relations between people and the world and recognizes the variability among learners. Therefore, assessment practice is continuously provided and individualized according to students’ specific needs and developmental levels (Orlando, 2011). In addition, highly rated translations can be developed into exemplars, which serve as model translations for students’ consideration. The longitudinal student translation corpus can not only allow teachers to continuously provide feedback on the translations of individual students but also achieve these translations as learning materials for students and teaching materials for teachers.

The longitudinal student translation corpus can also assist in the implementation of dynamic assessment. It can be used to develop an interventionist approach: computerized dynamic assessment. Persistent difficulties identified in students’ performance serve as the basis for developing prompts along a continuum of mediation. First, given the diversity of text types and a large number of learner translators, the corpus constitutes a wealth of learner data. This rich data provide an empirical basis for identifying the characteristics of student translation as students develop their translation competence throughout different periods. Second, the corpus comprises translated texts of the same translator over 2 years, enabling educators to track the developmental curve of the translation competence of their students at the individual level. The developmental curve can also be explained by identifying the factors contributing to the acquisition of translation competence with the rich metadata in the corpus.

## Conclusion

As van Lier (2004) noted, educational practices are value-laden and value-driven. The ecological approach to translation assessment discussed in this paper has important implications for translation teaching and research. The spatial and temporal dimensions of the context in which translation is produced merit attention. The level of external mediation a student requires to complete a translation task reveals his or her developmental potential. Past experiences provide a basis for charting his or her development. The longitudinal student translation corpus design proposed in the present paper makes it feasible to implement the ecological approach to assessment feedback, which constitutes an alternative to the traditional contrastive approach to translation assessment. Traditionally, student translators were evaluated against professional translators, assuming there is one ideal standard for translation learning. While translation education aims to train students to become professional translators, education *in situ* seeks to promote personal growth. The gap between achieved learning and the ideal standard, though answering the question “where to go,” could demotivate student translators at the low-intermediate levels. Before students meet the ideal standard, their learning performance will be defined as deficient. The ecological approach to assessment feedback addresses two problems of the traditional approach. First, it reiterates the developmental function of assessment and considers it as a tool that aids learning rather than a mere grading system (Galán-Mañas and Albir, 2015). Second, it shifts the focus from the assessment of performance to the assessment of learning and development by adding a temporal dimension. The objective of assessment feedback in education is to assess what a student has learned at the end of a given period. Students’ initial performance provides a benchmark against which their progress during the program can be measured. Using the initial state as the comparison benchmark would result in a qualitatively different portrait of the developmental curve of translation competence.

The paper has suggested ways forward for future assessment feedback practice and research in translation education. The focus of future assessment feedback may shift from current levels of performance to achieved development and learning potential, as well as the influencing contextual factors. There is a constant critical evaluation of the assessment feedback practice to check whether it has fulfilled the educational aim of promoting student development. Similarly, future research on assessment feedback in translation education may give due consideration to the complex contextual factors that contribute to translation competence development. Longitudinal research designs can be readily integrated with qualitative methods such as ethnographies and case studies.

The longitudinal student translation corpus to be compiled has the potential for technological innovations. The corpus is potentially helpful in developing an Automatic Translation Evaluation (ATE) system. The corpus data can be used to train machine learning with its translation divided into proficiency levels and annotated with various kinds of assessment feedback. To date, some machine translation metrics (e.g., BLEU, TER, and BERTSCORE) have been trialed in evaluating students’ translation and interpretation (Chung, 2020; Han and Lu, 2021). By machine learning techniques, a large quantity of student data can be used to enhance the accuracy of ATE. The platform under construction and the corpus to be compiled have the potential to contribute to the advancement of machine learning.

## Data availability statement

The original contributions presented in the study are included in the article/supplementary material, further inquiries can be directed to the corresponding author.

## Author contributions

DM, CZ and MHC contributed to the conception and design of the study and wrote and revised the manuscript. EM contributed to addressing the technical aspects of corups construction and reviewed the manuscript. All authors contributed to the article and approved the submitted version.

## Funding

This work was supported by Guangdong Provincial Social Science Fund (grant number #GD21YWY04) and Center for Translation Studies at Guangdong University of Foreign Studies (grant number #CTSZB201901).

## Conflict of interest

The authors declare that the research was conducted in the absence of any commercial or financial relationships that could be construed as a potential conflict of interest.

## Publisher’s note

All claims expressed in this article are solely those of the authors and do not necessarily represent those of their affiliated organizations, or those of the publisher, the editors and the reviewers. Any product that may be evaluated in this article, or claim that may be made by its manufacturer, is not guaranteed or endorsed by the publisher.
